# Effect of Epidermal Growth Factor and 6-Dimethylaminopurine on In Vitro Maturation and Artificial Activation of Spix’s Yellow-Toothed Cavy (*Galea spixii* Wagler, 1831) Oocytes

**DOI:** 10.3390/ani15162403

**Published:** 2025-08-15

**Authors:** Leonardo V. C. Aquino, Samara L. Olindo, Yara L. F. Silva, Vinícius D. Silva, Lhara R. M. Oliveira, Moacir F. Oliveira, Alexsandra F. Pereira

**Affiliations:** 1 Laboratory of Animal Biotechnology, Federal Rural University of the Semi-Arid, Mossoró 59625-900, RN, Brazil; leonardovt@live.com (L.V.C.A.); samara.lima@alunos.ufersa.edu.br (S.L.O.); yara.silva@alunos.ufersa.edu.br (Y.L.F.S.); viniciusd647@gmail.com (V.D.S.); lharagirs@hotmail.com (L.R.M.O.); 2 Center of Multiplication of Wild Animals, Federal Rural University of the Semi-Arid, Mossoró 59625-900, RN, Brazil; moacir@ufersa.edu.br

**Keywords:** wildlife, neotropical rodents, ex situ conservation, in vitro embryo production, oocyte viability, embryonic development

## Abstract

The successful application of assisted reproductive technologies (ARTs), such as in vitro maturation (IVM) and artificial oocyte activation, requires species-specific adaptations. Although these methods are routinely used in laboratory rodents, their use in wild or non-model species remains limited, such as the Spix’s yellow-toothed cavy, a Neotropical species of ecological and reproductive interest. This study evaluated the effects of different concentrations of epidermal growth factor (EGF; 10 or 50 ng/mL) on IVM (Experiment 1) and of 6-dimethylaminopurine (6-DMAP) on artificial oocyte activation (Experiment 2). EGF at 10 ng/mL (93.8% ± 1.6; 84.9% ± 0.7) promoted greater viability and less apoptosis in *cumulus* cells, compared to 50 ng/mL (83.0% ± 1.6; 78.9% ± 2.7), maintaining *cumulus* expansion, ultrastructural integrity, and better morphometric quality of oocytes. Thus, this concentration was used in the next step, where oocytes were activated with or without 6-DMAP. After five days, the presence of 6-DMAP increased cleavage rates (69.3% ± 5.0) compared to activation without the compound (53.5% ± 3.5), without significantly affecting morula formation (13.2% ± 3.1 to 17.3% ± 2.9). It is concluded that EGF improves the oocyte microenvironment, while 6-DMAP enhances cleavage, with these being the initial steps in the development of ARTs for Spix’s yellow-toothed cavy.

## Simple Summary

In vitro maturation (IVM) and artificial oocyte activation are crucial steps in the development of assisted reproductive technologies (ARTs), including somatic cell nuclear transfer. While these techniques are well-established in laboratory rodents, such as mice and rats, they remain underdeveloped in wild species, like Spix’s yellow-toothed cavy. Advancing ARTs in this species could support its conservation and help preserve biodiversity. This study evaluated the effects of two concentrations of epidermal growth factor (EGF; 10 or 50 ng/mL) in IVM medium, as well as the use of 6-dimethylaminopurine (6-DMAP) during artificial oocyte activation. Results showed that 10 ng/mL EGF enhanced *cumulus* cell viability and improved oocyte morphometric quality compared to 50 ng/mL. Although 6-DMAP positively influenced cleavage rates, it did not significantly affect morula formation. In conclusion, 10 ng/mL EGF is recommended for IVM in Spix’s yellow-toothed cavy oocytes, and 6-DMAP shows potential to support early embryonic development, contributing to the establishment of ART protocols for this species.

## 1. Introduction

The Spix’s yellow-toothed cavy (*Galea spixii* Wagler, 1831) is a Neotropical rodent with predominantly diurnal and crepuscular habits, whose diet is based on a diverse range of plant species [1]. Its natural geographic distribution includes the Caatinga biome, extending to other regions of Brazil, Paraguay, and the eastern slopes of the Andes Mountains [2]. This broad distribution reflects its remarkable ecological adaptability, granting it a relevant functional role in dry forest ecosystems, particularly through its contributions to seed dispersal, soil aeration, and trophic chain regulation [3,4].

In addition to its ecological importance, Spix’s yellow-toothed cavy exhibits reproductive traits that qualify it as a promising experimental model for the development of assisted reproductive technologies (ARTs). The species has a continuous polyestrous cycle with an average duration of approximately 14 days, as well as a gestation period of around 59 days, typically producing litters of one to two offspring [3]. These parameters, combined with its ease of management in captivity, due to its small body size, high metabolic rate, physiological adaptability, and consistent reproductive performance [5], reinforce its potential as a surrogate model for the development and standardization of protocols applicable to the conservation of phylogenetically related species, particularly those classified as threatened with extinction.

Among these vulnerable species are representatives of the suborder Hystricomorph, such as *Ctenomys ibicuiensis* and *Ctenomys flamarioni*, both classified as endangered and whose populations present significant logistical challenges for conducting reproductive studies [6]. In this context, the use of Spix’s yellow-toothed cavy as a model species represents a viable, ethical, and translational alternative, providing a solid foundation for the development of reproductive approaches that can later be adapted to these more sensitive taxa.

Although Spix’s yellow-toothed cavy is still considered a species with a stable population according to the International Union for Conservation of Nature (IUCN) [7], its increasing exposure to anthropogenic pressures, such as natural habitat fragmentation and illegal hunting, also raises concerns about its medium- and long-term sustainability and resilience. In this context, investing in research focused on its reproductive biology while populations remain stable represents a preventive and proactive strategy, enabling the early development of reproductive biotechnologies and genetic management protocols that may be more effectively applied in future scenarios of population decline or extinction risk.

Among wildlife conservation strategies, ARTs have gained significant attention [8]. One such ART is in vitro maturation (IVM) combined with artificial oocyte activation, both of which are essential steps in the development of techniques, such as somatic cell nuclear transfer (SCNT) [9]. With the advent of nuclear reprogramming—in which the epigenetic landscape of a somatic cell is altered by the remarkable reprogramming capacity of an enucleated oocyte to initiate embryonic development [10]—SCNT has emerged as a powerful biotechnological tool for the multiplication, conservation, and scientific study of reproductive biology in both laboratory and wild rodents [11].

However, the successful application of SCNT, including IVM and artificial oocyte activation, depends on species-specific adaptations [12]. Although these techniques are well established in laboratory rodents, their application in wild or non-model species remains limited and underexplored. Regarding IVM, it is clear that optimized protocols are essential, as oocyte development depends on factors such as temperature [13], duration [14], and the composition and supplementation of the culture medium [9] to acquire meiotic competence. In in vitro culture systems for both laboratory and wild rodents, epidermal growth factor (EGF) is frequently included due to its role as 1 of the 13 families of secondary proteins that regulate *cumulus* cell expansion [15,16]. These *cumulus* cells are crucial for signaling the resumption of meiosis I, facilitating first polar body extrusion, aligning the metaphase plate, and promoting cytoplasmic maturation [17,18]. Studies in other rodent species have demonstrated that EGF concentrations ranging from 10 to 50 ng/mL increase IVM efficiency [9,19], whereas higher doses may negatively affect oocyte quality and maturation success. Therefore, evaluating the effects of EGF concentration during IVM of Spix’s yellow-toothed cavy oocytes appears to be an important step in establishing this technical stage in the species.

It is essential to emphasize that, in addition to the oocyte undergoing IVM, the sensitivity of embryonic development during artificial activation is a crucial factor [10]. In both laboratory and wild rodents, a protocol combining 10 mM strontium chloride (SrCl_2_) with 5 μg/mL cytochalasin B (CB) for 6 h has been used to artificially activate oocytes of the red-rumped agouti, resulting in a morula formation rate of 25% [9]. Although blastocysts were not observed, these results suggest the potential for optimizing embryo production protocols for this genus. In contrast, artificial activation protocols in laboratory rodents have demonstrated greater efficiency, with blastocyst rates varying depending on the methods applied. For example, Ma et al. [20] reported two commonly used murine protocols: the first, using 10 mM SrCl_2_ and 5 μg/mL CB for 6 h, achieved a blastocyst rate of 56.3%; the second, a more effective protocol, applied 10 mM SrCl_2_ for 2.5 h followed by 2 mM 6-dimethylaminopurine (6-DMAP) and 5 μg/mL CB for 3.5 h, achieving a blastocyst rate of 60.1%. These findings highlight that artificial activation efficiency may be species-dependent, which reinforces the need to adapt and validate these protocols specifically for Spix’s yellow-toothed cavy in the context of its reproductive technologies.

Therefore, this study aimed to evaluate the effects of two EGF concentrations (10 or 50 ng/mL) in IVM medium and the use of 6-DMAP during the artificial activation of Spix’s yellow-toothed cavy oocytes. To this end, we assessed the quality of the microenvironment and matured oocytes by analyzing *cumulus* cell expansion, ultrastructure, viability, and apoptosis levels, as well as morphometric parameters, first polar body extrusion, nuclear stage, and embryonic development kinetics.

## 2. Materials and Methods

### 2.1. Chemicals and Media

Unless otherwise stated, all chemicals and media were purchased from Sigma-Aldrich (St. Louis, MO, USA) and Gibco-BRL (Carlsbad, CA, USA).

### 2.2. Bioethics and Animals

The Animal Use Ethics Committee of the Federal Rural University of Semi-Arid (CEUA/UFERSA, no. 23091.010566/2017-20) and the Chico Mendes Institute for Biodiversity Conservation (no. 60428-1) approved all experimental procedures. Nineteen healthy adult females, aged one year and weighing 250–350 g, were used in the study. They were housed in a controlled environment with access to fruit and water at the Center of Multiplication of Wild Animals (CEMAS/UFERSA, Mossoró, RN, Brazil; 5°10′ S, 37°10′ W), which is registered in Brazil in the Brazilian Institute of Environment and Renewable Natural Resources (IBAMA, no. 1478912).

### 2.3. Collection, Transport of Ovaries, and Recovery of the Cumulus–Oocyte Complexes

Females were subjected to a 12 h fasting period prior to ovary collection. Anesthesia was induced using a combination of xylazine hydrochloride (Xilazin^®^ 2%, 1 mg/kg, Manufacturer Syntec, São Paulo, SP, Brazil) and ketamine hydrochloride (Ketamine^®^ 10%, 15 mg/kg, Vetnil, São Paulo, SP, Brazil). Euthanasia was then carried out via intravenous administration of potassium chloride (Chlorure de potassium^®^ 19.1%, 2.56 mEq/kg, Halexistar, Goiânia, GO, Brazil) following established protocols [21].

After ovary collection using surgical scissors, the ovaries were transported to the laboratory in a pre-warmed saline solution (0.9% NaCl, *w*/*v*) at 30 °C, supplemented with 0.05 mg/mL penicillin (*w*/*v*), and maintained for up to 30 min [13]. For *cumulus*–oocyte complex (COC) retrieval, the ovaries were transferred to in vitro culture plates (60 × 15 mm) containing oocyte recovery medium (ORM). The ORM consisted of tissue culture medium (TCM-199) supplemented with 0.2 mM sodium pyruvate (*w*/*v*), 1% antibiotic-antimycotic (*v*/*v*), and 10% fetal bovine serum (FBS, *v*/*v*).

Following ovarian slicing, oocytes were recovered and morphologically evaluated under a stereomicroscope based on *cumulus* cell compaction, the number of surrounding cell layers, and ooplasm homogeneity. COCs were classified into three categories [14]: Type A—oocytes surrounded by three or more layers of *cumulus* cells; Type B—oocytes with one to three layers of *cumulus* cells; and Type C—oocytes with few or no *cumulus* cells, including denuded oocytes. Only COCs classified as Type A and Type B were selected for use in the experiments.

### 2.4. Experimental Design

**Figure 1:**
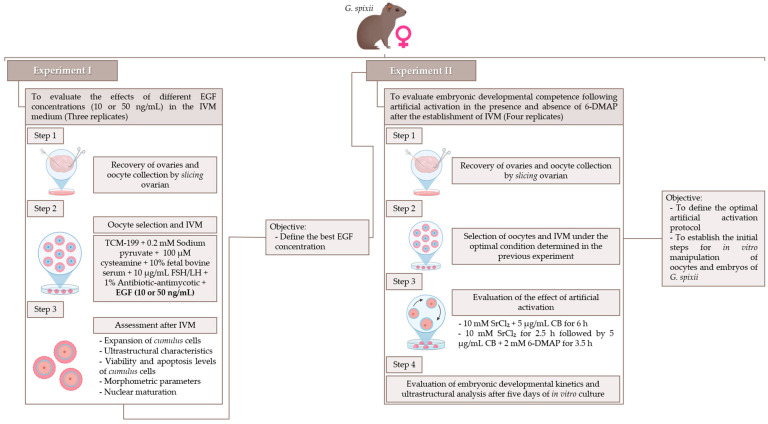
Experimental design of the steps involved in establishing in vitro manipulation conditions for oocytes and embryos of *G. spixii*.

Two experiments were conducted to evaluate the effects of different EGF concentrations (10 or 50 ng/mL) in the IVM medium (Experiment I) and the influence of 6-DMAP on artificial oocyte activation (Experiment II) (Figure 1). In Experiment I, seven females were used across three replicates (with two to three animals per replicate) to evaluate the effect of EGF on the oocyte microenvironment and nuclear maturation. Recovered oocytes were randomly allocated to IVM media supplemented with one of two EGF concentrations: 10 ng/mL (EGF10 group) and 50 ng/mL (EGF50 group). The concentrations selected for this study were based on prior research in other South American rodent models, including guinea pigs (*Cavia porcellus*) and red-rumped agoutis (*Dasyprocta leporina*). These studies demonstrated that EGF supplementation at concentrations between 10 and 50 ng/mL effectively promoted oocyte maturation and improved subsequent embryonic development. Conversely, doses exceeding this range were associated with reduced developmental competence and lower overall efficiency in vitro [9,19]. Analyses focused on the expansion of *cumulus* cells and their ultrastructural characteristics using scanning electron microscopy (SEM). Additionally, the viability and apoptosis levels of *cumulus* cells were evaluated to assess the quality of the in vitro culture environment. Oocytes were also examined for morphometric parameters and nuclear maturation, the latter determined by the extrusion rate of the first polar body (1PB) and classification of the nuclear stage in mature structures.

Following the selection of the optimal EGF concentration, Experiment II was conducted to evaluate the developmental competence of oocytes based on their ability to progress to early embryonic stages after artificial activation. Twelve females were used across four replicates (three animals per replicate). Recovered oocytes were matured in vitro using the previously determined optimal EGF concentration, and their quality was assessed according to the parameters previously described. Oocytes exhibiting 1PB extrusion were selected, subdivided into groups, and subjected to one of two artificial activation protocols: 10 mM SrCl_2_ + 5 µg/mL CB for 6 h (SrCl_2_ + CB group), or 10 mM SrCl_2_ for 2.5 h followed by 5 µg/mL CB + 2 mM 6-DMAP for 3.5 h (SrCl_2_ + CB + 6-DMAP group). In this study, 6-DMAP was evaluated during artificial activation using a standard protocol employed in the artificial activation of murine oocytes [20]. Finally, embryonic culture was conducted in a simple, optimized, and potassium-enriched medium (KSOM) for five days [19,22]. During this period, we monitored the kinetics of embryonic development and performed ultrastructural analyses to assess cleavage and embryo production rates.

### 2.5. In Vitro Maturation of Oocytes

For the oocyte IVM, recovered COCs were initially washed three times in ORM, followed by two additional washes in the IVM medium. Groups of approximately 20–25 oocytes were then cultured in 100 µL droplets of maturation medium, covered with mineral oil, and incubated at 38.5 °C in an atmosphere of 6.5% CO_2_ [23]. The maturation medium consisted of ORM supplemented with 0.1 mM cysteamine and 10 mIU/mL FSH/LH (Pluset^®^, Hertape-Calier, Barcelona, Spain) [9]. EGF was added to the IVM medium at concentrations of 10 or 50 ng/mL. After 24 h, mature structures were evaluated according to the parameters described in the experimental design.

### 2.6. Assessments of the Microenvironment and Oocyte Maturation

#### 2.6.1. Expansion of Cumulus Cells

Matured oocytes were evaluated for *cumulus* cell expansion under a stereomicroscope, following the classification method described by Zhao et al. [24]. COCs were scored on a scale from 0 to 4, as follows: Score 0—no observable expansion; Score 1—minimal expansion, with rounded *cumulus* cells predominantly adhered to the substrate; Score 2—expansion observed in the outer layers of *cumulus* cells, forming two distinct layers; Score 3—expansion of all layers except the corona radiata, forming more than three layers; Score 4—full expansion of both the *cumulus oophorus* and *corona radiata* cell complexes.

Moreover, the *cumulus* expansion index (CEI) was calculated for each EGF concentration tested. This index represents the mean expansion score within each group and was determined using the formula proposed by Fagbohun and Downs [25]. Groups with a CEI value approaching 0 were indicative of minimal *cumulus* expansion and suboptimal in vitro culture conditions. In contrast, values approaching 4 reflected complete expansion and were associated with higher oocyte quality [25].

#### 2.6.2. Ultrastructure Assessment by SEM

Matured oocytes previously exposed to different concentrations of EGF, as well as zygotes, were analyzed by scanning electron microscopy (SEM) to assess their cellular quality. Sample preparation followed the protocol described by Li et al. [26], with minor modifications. Briefly, the oocytes were initially fixed in 2.5% glutaraldehyde diluted in phosphate-buffered saline (PBS; pH 7.2) at 4 °C for 2 h.

Specifically for the zygotes, a preliminary step was performed to remove the zona pellucida prior to fixation. To accomplish this, the zygotes were washed in ORM-HEPES medium (25 mM HEPES; pH 2.5) until complete digestion of the zona pellucida and then fixed in glutaraldehyde, as previously described. After fixation, all samples (oocytes or zygotes) were washed twice for 5 min each in 2% cacodylate buffer and subsequently post-fixed in 1% osmium tetroxide for 2 h at room temperature (25 °C).

Following fixation, the cells (oocytes or zygotes) were washed again in PBS and dehydrated in a graded ethanol series (7.5%, 15%, 30%, 50%, 70%, 90%, and 100%) for 5 min at each concentration. After dehydration, the structures (oocytes or zygotes) were mounted on glass slides, gold-sputtered, and examined under a scanning electron microscope (TESCAN VEGA3; Tescan Analytics, Fuveau, Bouches-du-Rhône, France). This approach enabled a detailed analysis of the surface morphology of the evaluated structures, allowing for the observation of the structural integrity of oocytes and zygotes, the arrangement of *cumulus* cells, the texture and continuity of the zona pellucida, as well as the characteristics of the plasma membrane. These parameters provided relevant insights for assessing cellular quality and identifying possible morphological alterations resulting from the different experimental treatments applied.

#### 2.6.3. Viability and Apoptotic Levels of Cumulus Cells

Matured oocytes were denuded by mechanical pipetting in ORM containing 0.1% hyaluronidase (*w*/*v*). Next, 50 µL of the cell suspension was collected and stained with Trypan blue (0.2%) diluted in PBS [9]. The *cumulus* cell suspension was then analyzed for viability using a Neubauer chamber. Non-viable cells (dead; stained blue) were distinguished from viable cells (alive; colorless), based on the rupture of the cell membrane. The percentage of viable cells was calculated using the formula: number of viable cells/total number of cells quantified × 100.

The apoptotic levels of *cumulus* cells were determined using fluorescent dyes, following the method described by Rodrigues et al. [27] with modifications. For this analysis, a 50 µL aliquot of the suspension containing *cumulus* cells from previously denuded oocytes was collected. The cells were stained with ethidium bromide (5 µg/mL) and acridine orange (1 µg/mL), diluted in 8 µL of PBS. After the staining procedure, the sample was transferred to a glass slide and examined under a fluorescence microscope to assess the stages of cellular apoptosis. Through differential dye uptake, chromatin condensation in the cell nucleus was identified [28]. Based on fluorescence patterns, cells were classified into four categories: (i) viable cells, exhibiting uniform green fluorescence; (ii) early apoptosis, indicated by non-uniform green staining of the nucleus; (iii) late apoptosis, characterized by uniform orange nuclear fluorescence; and (iv) necrosis, marked by intense red fluorescence in the nucleus. This classification followed the criteria described by Aquino et al. [29]. For this, a fluorescence microscope (Olympus BX51TF, Tokyo, Japan) was used with an absorbance of 480 nm. Finally, images were saved, and 300 cells were counted for each replicate using Image J 7.1 (National Institutes of Health, Wellesley, MA, USA) software at 20× magnification.

#### 2.6.4. Morphometric and 1PB Evaluation of Oocytes

Morphometric analyses involved the morphometric evaluation of oocytes with and without the presence of 1PB after 24 h of IVM. Images were captured at 20× magnification using an inverted microscope (Leipzig IMx, Phoenix Optics, Leipzing, Germany). Pixel distances were converted using ImageJ 1.54 software to determine the outer oocyte diameter (OOD), inner oocyte diameter (IOD), zona pellucida thickness (ZPT), and ooplasm diameter (OD) [30]. Based on these parameters, the following measurements were calculated using mathematical formulas: space perivitelline diameter (SPD; OID—OD), internal oocyte area (IOA; π × (IOD/2)^2^), ooplasm area (OA; π × (OD/2)^2^), and perivitelline space area (PSA; IOA—OA) [31].

#### 2.6.5. Nuclear Stage Assessment

The nuclear stage of the oocytes was determined by observing the extrusion of the first polar body (1PB), followed by fluorescent staining. Initially, the oocytes were fixed in 4% paraformaldehyde solution for 15 min. After fixation, they were washed twice in phosphate-buffered saline (PBS) supplemented with 0.4% bovine serum albumin (PBS-BSA). Subsequently, nuclear staining was performed using 10 µg/mL of Hoechst 33,342 for 15 min. For this, a stock solution of Hoechst (5 mg/mL) was diluted in 1.5 mL of ORM to reach the desired final concentration. After staining, the oocytes were washed twice again in PBS-BSA to remove excess Hoechst 33,342 and mounted on glass slides. Fluorescence analysis was performed using a fluorescence microscope (Olympus BX51TF, Tokyo, Japan), and oocytes were considered mature when observed at the metaphase II stage [23]. The excitation wavelength for the fluorophore was 510 nm, and the emission was detected at 590 nm.

### 2.7. Artificial Activation, Ultrastructure Analysis, and Embryonic Kinetic Patterns

For artificial activation, denuded oocytes that had shown 1PB extrusion were used. The oocytes were first washed three times in ORM. Then, they were artificially activated in calcium-free Tyrode’s albumin lactate pyruvate (TALP) medium, supplemented with 10 mM SrCl_2_ and 5 µg/mL CB for 6 h at 38.5 °C and 6.5% CO_2_. For the 6-DMAP group, oocytes were initially incubated at 38.5 °C and 6.5% CO_2_ in TALP medium containing 10 mM SrCl_2_ for 2.5 h. After this period, the oocytes were washed three times in ORM and transferred to drops containing TALP medium with 5 µg/mL CB and 2 mM 6-DMAP for 3.5 h at 38.5 °C and 6.5% CO_2_, following the protocol described by Ma et al. [20]. The TALP medium was composed of salts, 10 mM sodium lactate (*v*/*v*), 3 mg/mL bovine serum albumin fraction V (*w*/*v*), and 1% antibiotic-antimycotic (*v*/*v*).

After artificial activation, presumed zygotes were rewashed in ORM and placed in 60 µL drops containing KSOM, which was supplemented with 1% essential amino acids, 0.5% non-essential amino acids, and 15 mM BSA. The structures were cultured for five days, with D0 defined as the day of artificial activation. On D2, cleavages were identified, and the rate for each group was recorded. Additionally, on D4 and D5, the production rates of morulae and blastocysts were observed.

Furthermore, during embryonic culture, one structure at each stage of embryonic development was recovered, fixed, and processed as previously described for ultrastructural evaluation by SEM of oocytes.

### 2.8. Statistical Analysis

All data were expressed as the mean ± standard error and were analyzed using StatView 5.0 (SAS Institute Inc, Cary, NC, USA). The normality of the data was verified using the Shapiro–Wilk test, while homoscedasticity was verified using Levene’s test. After confirming the normality and homoscedasticity of the data, variables related to *cumulus* cell expansion, viability and apoptotic levels of *cumulus* cells, presence of the 1PB, nuclear stage, and embryonic development rates were analyzed using Fisher’s exact test. CEI data and oocyte morphometric analyses were evaluated by analysis of variance (ANOVA), followed by Tukey’s test. Differences were considered statistically significant when the *p* < 0.05.

## 3. Results

**Figure 2:**
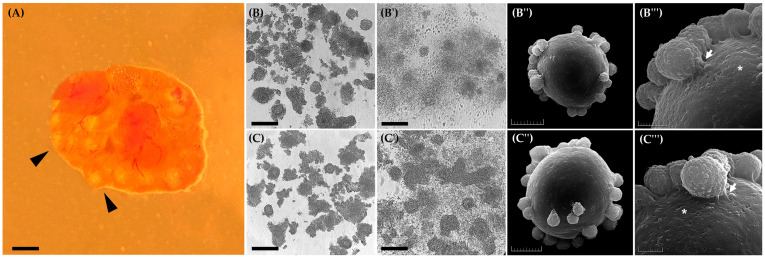
Representation of Spix’s yellow-toothed cavy ovaries and oocytes before and after IVM with EGF as a supplement to evaluate oocyte competence acquisition. (**A**) Ovary containing follicles (arrowhead) before processing by slicing. (**B**,**B′**) Immature Type I and II oocytes and *cumulus* cell expansion after 24 h of IVM with 10 ng/mL of EGF. (**B**″,**B‴**) Ultrastructural analysis of oocyte after IVM with 10 ng/mL EGF, showing transzonal projections from *cumulus* cells (arrow) and zona pellucida pores (asterisk). (**C**,**C′**) Immature Type I and II oocytes and *cumulus* cell expansion after 24 h of IVM with 50 ng/mL of EGF. (**C**″,**C‴**) Ultrastructural analysis of oocyte after IVM with 50 ng/mL EGF, showing transzonal projections from *cumulus* cells (arrow) and zona pellucida pores (asterisk). Scale bar = 100 µm (**A**); 50 µm, mag. 20× (**B**,**B’**,**C**,**C’**); 20 µm (**B**″,**C**″); 5 µm (**B‴**,**C‴**).

After slicing 38 ovaries (Figure 2A), the number of oocytes recovered and their morphological classification are detailed in Table 1. This table shows the proportion of each oocyte type obtained from the ovaries used in this study.

### 3.1. Experiment I: Effect of EGF on Cumulus Cells Expansion and Ultrastructural Visualization of Oocytes

Figure 2 shows immature oocytes before (Figure 2B,C) and after (Figure 2B′,C′) IVM with 10 and 50 ng/mL of EGF. It was observed that after the IVM process, neither concentration of EGF significantly influenced the different degrees of *cumulus* cell expansion (Table 2; *p* > 0.05). However, it is essential to note that 10 ng/mL and 50 ng/mL EGF resulted in more than 40% of the evaluated oocytes achieving a score of 4. Additionally, this result was considered positive, as the CEI analysis revealed oocytes with indices of up to 3.4, where a score of 4 represents the maximum value on the scale used to determine oocyte quality in this study (Table 2).

The ultrastructural analysis of oocytes matured in vitro with 10 ng/mL or 50 ng/mL of EGF (Figure 2B″,C″) revealed a clear adhesion of *cumulus* cells to the oocyte surface, forming densely organized layers around the zona pellucida (Figure 2B‴,C‴). In both groups, transzonal cytoplasmic projections crossing the zona pellucida were observed, indicating the maintenance of bidirectional communication pathways between the oocyte and *cumulus* cells, an essential feature for the exchange of signals and nutrients during maturation. Furthermore, no relevant morphological differences were identified between the treated groups, as the oocytes preserved the integrity of the zona pellucida, without signs of structural or surface disorganization. Additionally, the presence of small depressions or pores on the zona pellucida surface was observed, which may be associated with dynamic processes occurring during oocyte maturation (Figure 2B‴,C‴).

### 3.2. Experiment I: EGF Effect on Viability and Apoptotic Levels of Cumulus Cells

**Figure 3:**
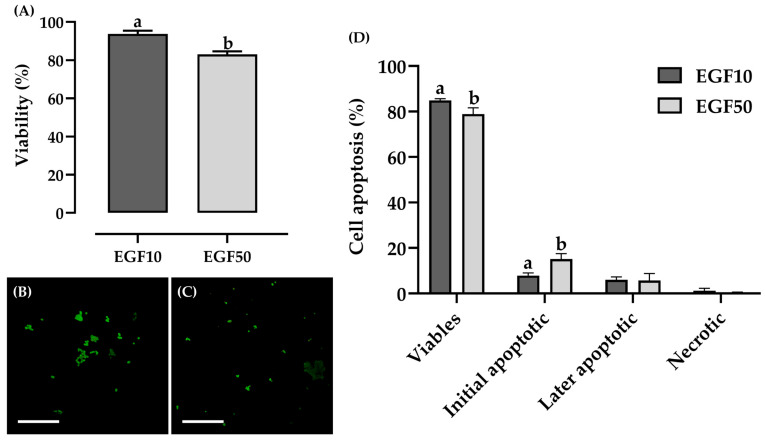
Assessment of viability and apoptotic levels of *cumulus* cells after 24 h of IVM of Spix’s yellow-toothed cavy oocytes. (**A**) Percentage of cell viability by Trypan blue assay. (**B**) Representation of the apoptotic assay in *cumulus* cells under IVM medium supplemented with 10 ng/mL EGF. (**C**) Representation of the apoptotic assay in *cumulus* cells under IVM medium supplemented with 50 ng/mL EGF. (**D**) Rate of apoptotic levels of *cumulus* cells under 10 and 50 ng/mL EGF. The results are expressed as mean ± standard error. a,b Values with different lowercase letters within columns significantly differ (*p* < 0.05). Scale bar = 50 µm (**B**,**C**), mag. 20×. Three repetitions.

Cell viability was assessed using the Trypan blue assay (Figure 3A). It was observed that the IVM medium supplemented with 10 ng/mL EGF (93.8% ± 1.6; 982/1047) ensured greater *cumulus* cell viability compared to the medium with 50 ng/mL EGF (83.0% ± 1.6; 643/775). In addition to cell viability, the apoptotic levels of *cumulus* cells were analyzed (Figure 3B,C). It was observed that in the EGF10 group (84.9% ± 0.7; 764/900), the cells were more viable compared to the EGF50 group (78.9% ± 2.7; 710/900) (Figure 3D). This difference may be attributed to the higher number of cells in early apoptosis observed in the EGF50 group (15.1% ± 2.4; 136/900) compared to the EGF10 group (7.9% ± 1.1; 71/900) after 24 h of IVM (Figure 3D). No significant differences were observed later between the groups regarding the number of cells in later stages of apoptosis and necrosis (Figure 3D).

### 3.3. Experiment I: Effect of EGF on Morphometric Parameters and Nuclear Maturation

**Figure 4:**
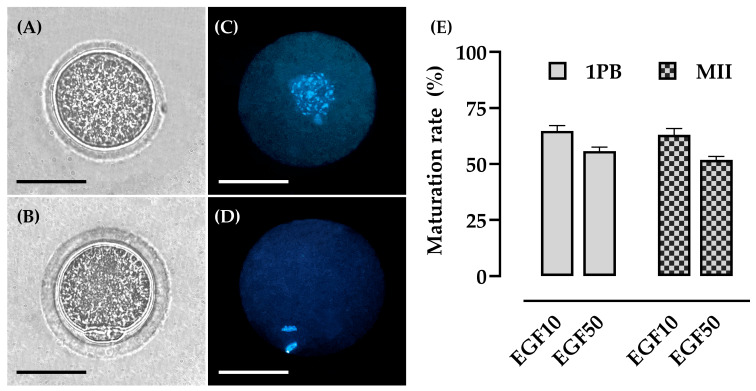
Representative image of Spix’s yellow-toothed cavy oocytes before and after 24 h of IVM containing different concentrations of EGF. (**A**) Immature oocyte. (**B**) Oocyte after 1PB extrusion. (**C**) Oocyte in germinal vesicle breakdown (GVBD) configuration. (**D**) Oocyte in metaphase II. (**E**) Percentage of oocyte rate after nuclear maturation assessment. The results are expressed as mean ± standard error. Scale bar = 100 µm, mag. 20× (**A**,**B**,**C**,**D**). Three repetitions.

After 24 h of IVM, a morphometric evaluation was performed on immature oocytes and those showing extrusion of the 1PB (Figure 4A,B). The results are presented in Table 3. It was observed that oocytes matured in IVM medium supplemented with 10 ng/mL EGF had a larger diameter compared to those supplemented with 50 ng/mL EGF, regarding IOD (78.8 ± 5.2 μm vs. 74.9 ± 2.6 μm), ZPT (9.4 ± 0.8 μm vs. 8.5 ± 0.9 μm), and OD (69.6 ± 4.2 μm vs. 64.8 ± 2.8 μm). Consequently, oocytes matured with 10 ng/mL EGF exhibited a larger IOA (15,502 ± 2466 μm^2^ vs. 13,877 ± 957 μm^2^) and OA (12,058 ± 1630 μm^2^ vs. 10,412 ± 901 μm^2^) compared to those with 50 ng/mL EGF.

Regarding the evaluation of nuclear maturation, the extrusion rate of the 1PB was observed, as well as the analysis of the nuclear stage of oocytes matured in vitro (Figure 4B–D). In this context, it was observed that there was no difference between oocytes subjected to in vitro maturation with 10 ng/mL EGF (64.8% ± 2.4; 35/54) and 50 ng/mL EGF (55.8% ± 1.7; 29/52) after 1PB extrusion. Additionally, no differences were observed between the groups after evaluating the nuclear stage (63.0% ± 2.9; 34/54 vs. 51.9% ± 1.5; 27/52) (Figure 4D). Although this was the case, it is important to note that the results obtained in this study were promising and represent a significant step towards implementing in vitro techniques in this species.

### 3.4. Experiment II: Assessment of the Microenvironment and Quality of Oocytes After IVM

Considering the benefits of EGF10 observed in the previous experiment, oocytes used in the second experiment were subjected to IVM in medium supplemented with 10 ng/mL EGF. To ensure the appropriate microenvironment and nuclear maturation of the oocytes that were artificially activated, cells were analyzed across four replications for expansion grade, viability, and apoptotic levels of *cumulus* cells. Additionally, 1PB extrusion was observed, and cells exhibiting this structure were subjected to artificial activation to evaluate embryonic development rates over a five-day period.

The results regarding the grade of expansion of cumulus cells and CEI are presented in Table 4. Concerning viability, as assessed by the trypan blue assay, oocytes matured in the presence of 10 ng/mL EGF exhibited a viability rate greater than 90% (2970/3279). Additionally, 97% (878/900) of *cumulus* cells were viable following the apoptotic assay. Finally, regarding 1PB extrusion, oocytes matured with EGF10 showed a rate of 64% (146/227). These oocytes were then randomly subdivided to evaluate the effect of the presence or absence of 6-DMAP, as per the artificial activation protocol previously described in the experimental design.

### 3.5. Experiment II: Effect of 6-DMAP on Activation and Kinetics of Embryonic Development

**Figure 5:**
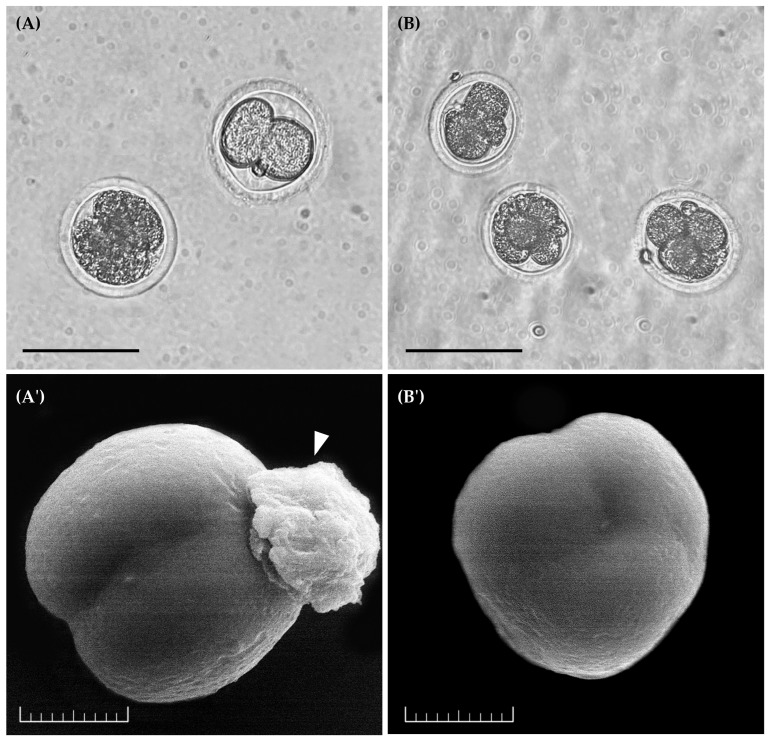
Representative image of cleaved zygotes after artificial activation and two days of embryonic culture. (**A**) Zygotes with 2 cleavages. (**B**) Zygotes with 3–7 cleavages. (**A′**) Ultrastructure of zygotes with two cleavages. The arrowhead indicates remains of the degraded zona pellucida after processing for visualization in SEM. (**B′**) Ultrastructure of cleavages containing three blastomeres. Scale bar = 100 µm, mag. 20× (**A**,**B**); 20 µm (**A′**,**B′**). Four repetitions.

After artificial activation, analysis of embryonic development kinetics and ultrastructure (Figure 5) revealed that the zygotes exhibited cleavage stages ranging from two cells to three to seven cells (Figure 5A,B). In addition, zona pellucida digestion in the zygotes was observed following SEM sample processing (Figure 5A′). The ultrastructural analysis of the zygotes revealed the outer surfaces of the blastomeres, which appeared spherical and showed clear signs of cell division (Figure 5A′,B′).

**Figure 6:**
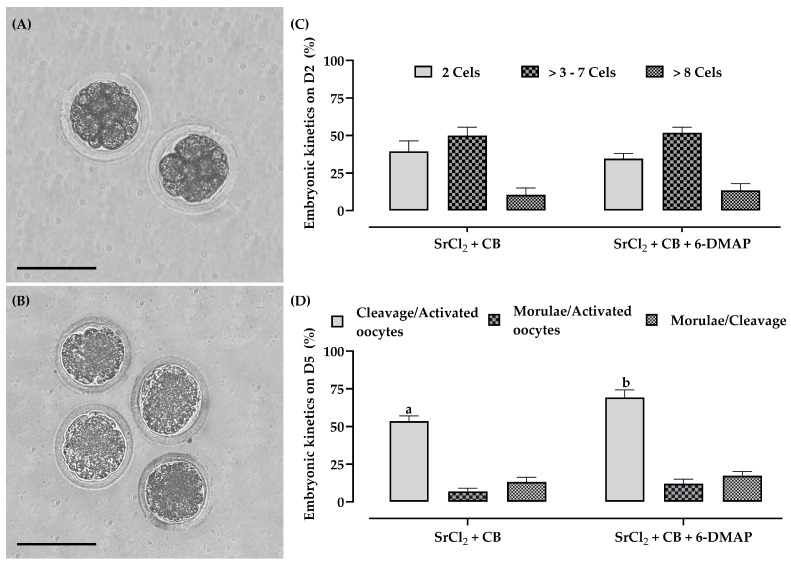
Embryonic development rate of artificially activated *G. spixii* oocytes. (**A**) Zygotes with cleavages > 8 cells after two days of embryonic culture. (**B**) Compacted morulae after five days of embryonic culture. (**C**) Embryonic cleavage rates after 48 h in vitro culture. (**D**) Cleavage and morulae formation rates after 120 h of embryonic development. Scale bar = 100 µm, mag. 20× (**A**,**B**). The results are expressed as mean ± standard error. a,b Values with different lowercase letters within columns significantly differ (*p* < 0.05).

During the evaluation of embryonic development kinetics over five days, artificially activated oocytes achieved cleavages beyond eight cells (Figure 6A). For the first time in this species, compacted morulae were also obtained (Figure 6B). Regarding embryonic development kinetics, 6-DMAP did not affect the number of cleavages in two-cell, three- to seven-cell, and >eight-cell zygotes (Figure 6C). However, the presence of 6-DMAP (69.3% ± 5.0; 52/75) positively influenced the total number of cleaved zygotes compared to the group without the secondary activator (53.5% ± 3.5; 38/71) (Figure 6D), resulting in the production of compacted morulae at rates of 13% ± 3.1 (5/38) in the group without 6-DMAP and 17% ± 2.9 (9/52) in the group supplemented with 6-DMAP (Figure 6D).

## 4. Discussion

We developed and refined in vitro manipulation strategies for Spix’s yellow-toothed cavy oocytes and embryos over seven experimental replicates, aiming to evaluate the effects of EGF supplementation at two concentrations (10 and 50 ng/mL) during the IVM process, as well as the impact of 6-DMAP during artificial activation. We analyzed the influence of EGF on the quality of the microenvironment and nuclear maturation of oocytes. EGF interacts with specific receptors that play essential roles in the endocrine, paracrine, and autocrine processes of oocytes and surrounding cells, positively influencing differentiation, communication, and nuclear and cytoplasmic maturation [17,18]. After selecting the optimal EGF concentration, we assessed the mature structures using an established artificial activation protocol for red-rumped agoutis, mice, and rats [9,20]. At this stage, we analyzed the influence of the presence or absence of 6-DMAP. This molecule inhibits serine/threonine kinases and seems to enhance the efficiency of artificial activation for greater in vitro embryo production [32,33].

Regarding *cumulus* cell expansion, our study observed that both EGF concentrations, 10 ng/mL and 50 ng/mL, resulted in more than 40% expansion of the *cumulus oophorus* complex and the corona radiata, achieving a Score of 4. This was subsequently justified by the CEI values, which ranged between 3.1 and 3.4. In contrast, a study by Praxedes et al. [9] with red-rumped agouti demonstrated that 10 ng/mL EGF in conjunction with 10 mIU/mL FSH resulted in a 31.8% ± 1.3 rate of oocytes with Score 3 and 15.9% ± 0.9 with Score 4, reaching a CEI of up to 3.6. This discrepancy may be attributed to the fact that, although phylogenetically close, there are intrinsic biological and reproductive differences between these species [34,35]. In Spix’s yellow-toothed cavies, EGF may be more efficiently utilized for *cumulus* cell expansion without affecting the CEI. In contrast, in red-rumped agouti, there may be lower efficiency in the activation of these molecules at this concentration. For example, in mice, when paracrine and juxtacrine components of the ovulatory signal were inactivated by knockout of EGF-like transmembrane peptides AREG and EREG [36,37], reduced efficiency in cumulus cell expansion and oocyte maturation was observed.

Another justification is that while FSH exerts an inhibitory effect on the initial resumption of meiosis, it induces the final stages of meiotic maturation [24]. This process is regulated by the expression levels of natriuretic peptide precursor type C (NPPC) in *cumulus* cells and the positive modulation of natriuretic peptide receptor 2 (NPR2), both of which are influenced by the actions of FSH and EGF. The oocytes in these species may respond differently to the treatments observed in each species [38], which could affect *cumulus* cell expansion. For example, in red-rumped agouti (*D. leporina*), a higher rate of Score 3 was observed compared to Score 4, and when compared to Spix’s yellow-toothed cavy. For this reason, we evaluated Spix’s yellow-toothed cavy oocytes in our study for their ultrastructural characteristics, viability, and apoptotic levels as quality indicators of the in vitro environment.

Regarding viability after Trypan blue staining, oocytes in medium supplemented with 10 ng/mL EGF had a higher rate of viable cells compared to 50 ng/mL EGF. Consequently, we performed an analysis of apoptotic levels of these cells using ethidium bromide and acridine orange assays. In this context, we observed that the medium supplemented with 50 ng/mL EGF showed a higher initial apoptosis index in *cumulus* cells compared to 10 ng/mL EGF. This result may reflect the metabolic activity of oocyte mitochondria, which are involved in the production of reactive oxygen species (ROS), leading to a higher incidence of apoptosis in *cumulus* cells [39]. Therefore, a more effective antioxidant supplementation than the cysteamine used in our study may be necessary.

Additionally, during the acquisition of meiotic competence of mouse oocytes in vitro, EGF promotes a significant and rapid increase in phosphorylation within the first 30 min of the event compared to oocytes matured in vivo [40]. This suggests that this metabolic activity may lead to increased ATP production, which in turn negatively affects the quality of *cumulus* cells through the subsequent synthesis of ROS molecules [41]. Consequently, this results in a lower cellular viability rate, as observed after Trypan blue staining and apoptosis assays. Another associated reason is that it has been demonstrated in vitro that *cumulus* cells can also be affected by the accumulation of glycosylations derived from transmembrane proteins (AREG, EREG, and BTC) in response to EGF [42,43]. Therefore, it is possible that an excess of these factors could cause significant damage to the *cumulus* cells of Spix’s yellow-toothed cavy oocytes.

Although supplementation with 50 ng/mL EGF reduced the viability rate of *cumulus* cells, our study did not observe any morphological changes from the SEM analysis. Our results revealed that oocytes matured under both EGF supplementation conditions maintained the necessary machinery for development and oocyte competence. This is justifiable as we could observe the highly specialized transzonal cytoplasmic projections of cumulus cells penetrating the zona pellucida of Spix’s yellow-toothed cavy oocytes [17]. This likely ensures the interface and intricate bidirectional communication between the microenvironment and the oocyte, which are crucial for providing the necessary nutrition and support for meiotic acquisition [17]. This result is corroborated by our study, where, in the initial attempt to perform IVM of Spix’s yellow-toothed cavy oocytes using EGF as a supplement, we observed nuclear maturation rates ranging from 55% to 64% for the extrusion of the 1PB, while 51% to 63% corresponded to the metaphase II nuclear stage. These rates are similar when compared to guinea pigs [13,19], paca (*Cuniculus paca*) [44], *D. prymnolopha* [45], and red-rumped agouti (*D. leporina*) [9].

In addition to the parameters analyzed, we performed, for the first time, morphometric evaluations on Spix’s yellow-toothed cavy oocytes regarding structures that exhibited or did not exhibit the extrusion of the 1PB after IVM with 10 or 50 ng/mL EGF. According to Saadeldin et al. [31], assessing oocyte morphometry can also be a decisive step in evaluating the quality and meiotic competence for adequate embryonic development following artificial activation. Thus, a larger ooplasm diameter (OD) and a smaller space perivitelline diameter (SPD) are expected to indicate better oocyte quality [31]. In our study, we initially observed that Spix’s yellow-toothed cavy oocytes exhibited a diameter ranging from 92.8 to 94.3 μm. Additionally, after IVM, oocytes matured in media containing 10 ng/mL EGF demonstrated a larger OD compared to those matured with 50 ng/mL EGF. However, no significant difference was observed in SPD. Notably, this result is interesting because, in in vivo systems, oocytes from small antral follicles are usually not responsive to EGF signaling cascades [46]. In contrast, COCs from Graafian follicles can respond to progressively increasing EGF levels, thereby enabling better embryonic development [47,48]. Therefore, the acquisition of EGF signaling capability by *cumulus* cells in relation to oocytes aligns with the mechanisms governing oocyte meiotic competence, and these processes are likely interrelated [48,49].

Based on the previous justifications, we selected 10 ng/mL EGF as the ideal concentration for use during the IVM of Spix’s yellow-toothed cavy oocytes. We considered this concentration for evaluating artificial activation in the presence or absence of 6-DMAP. Initially, regarding the artificial activation protocol for embryonic development, we chose 10 mM SrCl_2_, which can induce repetitive intracellular calcium releases, similar to what occurs during in vitro fertilization in mice and rats, which are laboratory rodents phylogenetically close to Spix’s yellow-toothed cavy. In these species, 10 mM SrCl_2_ mimics natural conditions, similar to the role of spermatozoa on the oocyte, providing an opportunity for the reconstruction of cloned embryos or in physiological studies [50,51]. Additionally, in our study, we also used 5 µg/mL CB. This compound is included during cloning and prevents the formation and extrusion of the second polar body, resulting in diploid embryos. In mice, its addition has significantly enhanced blastocyst development compared to artificial activation without this compound [20]. This occurs because, in diploid mouse embryos, a lower incidence of apoptosis is observed compared to haploid embryos [52]. We therefore hypothesized that its addition could enhance embryo quality in our experiment.

In our study, we also evaluated the presence or absence of 6-DMAP as a secondary activator. The addition of this compound was based on previous evidence showing that its inclusion in activation protocols in mice can significantly enhance artificial activation efficiency, achieving blastocyst rates of up to 60%. This effect is likely due to the reduction in meiosis-promoting factor (MPF) activity, thereby improving cell cycle progression efficiency. In our protocol, we combined 10 mM SrCl_2_ for 2.5 h followed by exposure to 5 µg/mL CB and 2 mM 6-DMAP for 3.5 h (SrCl_2_ + CB + 6-DMAP). We observed a higher overall cleavage rate compared to the control group (without 6-DMAP), although the compact morulae formation rate remained limited to 13% to 17%. This result may be related to the fact that, although the Spix’s yellow-toothed cavy is phylogenetically close to other laboratory and wild rodents, important gaps remain in our understanding of reproductive technologies applied to this species.

Therefore, implementing optimized protocols by adjusting gas composition, temperature, medium supplementation, and IVM duration could represent a viable alternative to improve this developmental step. This observation underscores the need for further investigations, especially considering that studies in mice have shown that treatment with chorionic gonadotropin (hCG), administered between days 13 and 18, can lead to artificial activation rates of 30% to 60% in mature oocytes [20].

Thus, future studies are essential to enable embryonic development beyond the morula stage, potentially reaching blastocyst formation and hatching. Although blastocysts were not observed under the in vitro conditions used in this study, the achievement of compact morulae represents a biologically relevant milestone for this species. This finding demonstrates that the combination of IVM and artificial activation was sufficient to support early key processes of embryonic development, such as cleavage, compaction, and cell polarization, steps that are tightly regulated and indicative of embryonic competence. Considering that there are no prior reports describing in vitro embryo production beyond early cleavage stages in *G. spixii*, our findings expand the application of reproductive biotechnologies in this Neotropical rodent.

Despite this limitation, the production of compact morulae represents a significant experimental advancement and provides a solid foundation for improving reproductive biotechnologies in wild rodents of the Hystricomorph suborder. Furthermore, this progress paves the way for future investigations involving nuclear transfer, embryo cryopreservation, and the development of embryo transfer protocols tailored to this species.

## 5. Conclusions

This was the first study to establish the complete sequence of in vitro manipulation steps for oocytes and embryos of Spix’s yellow-toothed cavy, with the aim of promoting species multiplication, conservation, and reproductive research. Our findings indicate that supplementation with 10 ng/mL of EGF during IVM is the most suitable condition, as it enhances *cumulus* cell viability and reduces apoptosis rates. Additionally, EGF10 improved the morphometric quality of oocytes and *cumulus* structures, achieving 60% nuclear maturation.

We also demonstrated, for the first time, the production of compact morulae using an artificial activation protocol commonly applied to laboratory rodents. Although the protocol still requires optimization to reach later embryonic stages, this represents a significant advancement for this species.

These results provide a foundational framework for the development of assisted reproductive technologies (ARTs) tailored to *G. spixii*, and they highlight the potential of this species as a model for reproductive biotechnology in Neotropical hystricomorph rodents. In a broader context, the methodologies and outcomes presented here may directly contribute to ex situ conservation programs, support the establishment of genetic resource banking, and inform comparative studies on ART development in other wild rodent species. Future efforts should focus on refining embryo culture systems, extending embryonic development to the blastocyst stage, and evaluating the feasibility of embryo cryopreservation and transfer in this species.

## Figures and Tables

**Table 1:** Number and classification of oocytes recovered from Spix’s yellow-toothed cavy ovaries.

Experiment	No. Ovaries	Classification of Immature Oocytes			Oocytes Type A/No. Ovaries	Oocytes Type B/No. Ovaries	Oocytes Type C/No. Ovaries	Total Oocytes/Ovaries
		Type A (%)	Type B (%)	Type C (%)				
I	14	52.7 ± 5.5 (78/148)	18.9 ± 3.7 (28/148)	28.4 ± 2.1 (42/148)	5.6 ± 1.6	2.0 ± 0.0	3.0 ± 0.5	10.7 ± 2.0
II	24	44.6 ± 5.0 (107/240)	50.0 ± 2.3 (120/240)	5.4 ± 3.2 (13/240)	4.5 ± 0.2	5.0 ± 0.8	0.5 ± 0.3	10.0 ± 0.4

Note: The results are expressed as mean ± standard error. Type (A): Oocytes that had three or more layers of *cumulus* cells; Type (B): Oocytes that had between 1 and 3 layers of *cumulus* cells; Type (C): Oocytes that had few or no *cumulus* cells or were naked, as described by Yao et al. [14]. Experiment I: three repetitions; Experiment II: four repetitions.

**Table 2:** Evaluation of the grade of *cumulus* cell expansion and CEI of Spix’s yellow-toothed cavy oocytes matured in IVM medium supplemented with two concentrations of EGF after 24 h.

Groups	No. Oocytes	Grade of *Cumulus* Cell Expansion (%)					CEI
		Score 0	Score 1	Score 2	Score 3	Score 4	
EGF10	54	-	1.8 ± 1.5 (1/54)	13.0 ± 1.2 (7/54)	29.6 ± 4.2 (16/54)	55.6 ± 2.6 (30/54)	3.4 ± 0.1
EGF50	52	-	9.6 ± 0.3 (5/52)	11.5 ± 3.5 (6/52)	34.6 ± 9.0 (18/52)	44.2 ± 7.5 (23/52)	3.1 ± 0.1
P	-	-	0.1093	1.000	0.6784	0.3314	0.120

Note: The results are expressed as mean ± standard error. The CEI (*cumulus* expansion index) was assessed according to a subjective scoring system, ranging from 0 to 4, where 0 indicated the absence of expansion and 4 indicated the expansion of all layers. The calculation of the average expansion was performed as described by Fagbohun and Downs [25]. According to the example—of 55 COCs, 20 COCs had a score of 0; 20 COCs had a score of 1; 10 COCs had a score of 2; 5 COCs had a score of 3; and 0 COCs had a score of 4—this group would present the CEI calculated: [(0  ×  20)  +  (1  ×  20)  +  (2  ×  10)  +  (3  ×  5)  +  (4  ×  0)]/55  =  1.0. Three repetitions.

**Table 3:** Morphometric evaluation of immature and mature Spix’s yellow-toothed cavy oocytes after 24 h of IVM supplemented with EGF.

Groups	1PB	No. Oocytes	OOD (μm)	IOD (μm)	ZPT (μm)	OD (μm)	SPD (μm)	IOA (μm^2^)	OA (μm^2^)	PSA (μm^2^)
EGF10	−	19	92.8 ± 4.5 ^a^	76.4 ± 2.9 ^ab^	9.3 ± 0.5 ^ab^	69.2 ± 2.4 ^a^	7.2 ± 2.3 ^a^	14,431 ± 1087 ^ab^	11,833 ± 805 ^ab^	2598 ± 840 ^a^
	+	35	95.7 ± 6.9 ^a^	78.8 ± 5.2 ^a^	9.4 ± 0.8 ^a^	69.6 ± 4.2 ^a^	9.2 ± 3.4 ^a^	15,502 ± 2466 ^a^	12,058 ± 1630 ^a^	3443 ± 1437 ^a^
EGF50	−	23	93.9 ± 0.2 ^a^	76.7 ± 2.6 ^ab^	8.9 ± 0.8 ^ab^	69.0 ± 3.3 ^a^	7.7 ± 2.7 ^a^	14,564 ± 1021 ^ab^	11,813 ± 1101 ^ab^	2751 ± 941 ^a^
	+	29	94.3 ± 1.7 ^a^	74.9 ± 2.6 ^b^	8.5 ± 0.9 ^b^	64.8 ± 2.8 ^b^	10.1 ± 2.7 ^a^	13,877 ± 957 ^b^	10,412 ± 901 ^b^	3464 ± 926 ^a^

Note: The results are expressed as mean ± standard error. ^a,b^ Values with different superscript letters within columns significantly differ (*p* < 0.05). OOD, outer oocyte diameter; IOD, inner oocyte diameter; ZPT, zona pellucida thickness; OD, ooplasm diameter; SPD, space perivitelline diameter; IOA, internal oocyte area; OA, ooplasm area; PSA, perivitelline space area. The morphometric calculation for each parameter evaluated on the oocytes was performed as described by Saadeldin et al. [31]. Three repetitions.

**Table 4:** Rate of expansion of *cumulus* cells and CEI of *G. spixii* oocytes after 24 h of IVM in medium supplemented with 10 ng/mL of EGF.

Group	No. Oocytes	Grade of *Cumulus* Cell Expansion (%)					CEI
		Score 0	Score 1	Score 2	Score 3	Score 4	
EGF10	54	1.3 ± 0.9 (3/227)	6.6 ± 1.2 (15/227)	21.1 ± 3.9 (48/227)	27.1 ± 3.4 (66/227)	41.9 ± 2.9 (95/227)	3.0 ± 0.03

Note: The results are expressed as mean ± standard error. The CEI (*cumulus* expansion index) was assessed according to a subjective scoring system, ranging from 0 to 4, where 0 indicated the absence of expansion and 4 indicated the expansion of all layers. The calculation of the average expansion was performed as described by Fagbohun and Downs [25]. According to the example—of 55 COCs, 20 COCs had a score of 0; 20 COCs had a score of 1; 10 COCs had a score of 2; 5 COCs had a score of 3; and 0 COCs had a score of 4—this group would present the CEI calculated: [(0  ×  20)  +  (1  ×  20)  +  (2  ×  10)  +  (3  ×  5)  +  (4  ×  0)]/55  =  1.0. Four repetitions.

## Data Availability

The data presented in this study are available upon request from the corresponding author.

## Abbreviations

The following abbreviations are used in this manuscript:

1PB	First polar body
6-DMAP	6-dimethylaminopurine
ARTs	Assisted reproductive technologies
BSA	BSA bovine serum albumin
CB	Cytochalasin B
CEI	*Cumulus* expansion index
CEMAS	Centre of Multiplication of Wild Animals
CEUA	Animal Use Ethics Committee
COCs	*Cumulus*–oocyte complexes
EGF	Epidermal growth factor
GVBD	Oocyte in the germinal vesicle breakdown
hCG	Chorionic gonadotropin hormones
IOA	Internal oocyte area
IOD	Inner oocyte diameter
IVM	In vitro maturation
KSOM	Simple, optimized, and potassium-enriched medium
MPF	Meiosis-promoting factor
OA	Ooplasm area
OD	Ooplasm diameter
OOD	Outer oocyte diameter
ORM	Oocyte recovery medium
PBS	Phosphate buffer solutions
PSA	Perivitelline space area
ROS	Reactive oxygen species
SCNT	Somatic cell nuclear transfer
SEM	Scanning electron microscopy
SPD	Space perivitelline diameter
SrCl_2_	Strontium chloride
TALP	Tyrode’s albumin lactate pyruvate
UFERSA	Federal Rural University of Semi-Arid
